# The role of timely initiation of antenatal care on protective dose tetanus toxoid immunization: the case of northern Ethiopia post natal mothers

**DOI:** 10.1186/s12884-018-1878-y

**Published:** 2018-06-15

**Authors:** Muhabaw Shumye Mihret, Miteku Andualem Limenih, Temesgen Worku Gudayu

**Affiliations:** Department of Midwifery, College of Medicine and Health Sciences, University of Gondar, Po. Box 196, Gondar, Ethiopia

**Keywords:** Debretabor, Immunization, Protective dose, Tetanus toxoid

## Abstract

**Background:**

Globally, tetanus toxoid protective dose immunization of the mothers is one of the strategies of maternal and neonatal tetanus prevention. Ethiopia has planned the national tetanus protection at birth coverage to reach 86% by the year 2015. However, there is still low coverage with less identified associated factors. Therefore; the purpose of this study was to assess tetanus toxoid protective dose immunization at last birth and associated factors among mothers who gave birth within one year prior to the study in Debretabor town, Northwest Ethiopia, 2016**.**

**Methods:**

A community based cross sectional study was conducted from May 1 to June 10 / 2016. A total of 511 mothers were included in the study. Structured questionnaire and checklists were used to collect the data. Face to face interview with cross checking documented record were employed. A systematic random sampling technique was used. The data were entered in to Epinfo version 7.0 and then exported to SPSS version 20.0 for analysis. Both bivariate and multivariable logistic regression model were fitted and crude and Adjusted Odds ratio with 95% confidence interval were computed. Finally, statistically significant association of variables was determined based on Adjusted Odds ratio with its 95% confidence interval and *p*-value ≤0.05.

**Result:**

The proportion of tetanus toxoid protective dose immunization among mothers was 56.2% (95% CI: 52–60%). In the multivariable analysis; formal education (AOR = 2.09; 95%CI: 1.12, 3.90), planned last pregnancy (AOR = 6.63; 95%CI: 2.36, 18.63), four or more antenatal care visits (AOR = 5.16; 95%CI: 2.93, 11.14), timely antenatal care visit (AOR = 4.29; 95%CI: 1.94, 9.49), and perceived good quality of service (AOR = 2.20; 95% CI: 1.26, 3.84) were positively associated with tetanus toxoid protective dose immunization.

**Conclusion:**

In this study, protective dose tetanus toxoid immunization is lower than the national target. Strengthening information education communication regarding tetanus and its prevention and encouraging timely initiation of and complete attendance of antenatal care is recommended.

**Electronic supplementary material:**

The online version of this article (10.1186/s12884-018-1878-y) contains supplementary material, which is available to authorized users.

## Background

Protected at birth (PAB) against tetanus is the proportion of births protected from tetanus infection at birth by tetanus toxoid protective dose immunization (TTPDI) of the mothers before birth [1, 2]. World Health Organization (WHO) recommends 5 consecutive doses of tetanus toxoid (TT) vaccination for child bearing age women (CBAW) per schedule to protect the birth against tetanus [3]. Tetanus is a highly fatal, non-communicable, toxin-mediated disease caused by Clostridium tetani bacteria [3, 4]. Women and their newborns are at high risk of acquiring tetanus related to the birthing process [5]. Globally, tetanus toxoid (TT) immunization of the mothers is one of the preventive strategies of maternal and neonatal tetanus (MNT) at birth [5–7].

Maternal and neonatal tetanus (MNT) is a public concern mainly accreditable to low maternal TT vaccination [8]. Fortunately, almost all developed countries have achieved maternal and neonatal tetanus (MNT) elimination prior to 1999 either by full coverage of TT immunization or by hygienic birth practice [9]. However, it has been a big deal in developing regions. Between 1999 and 2014, still about 33 million child bearing age women (CBAW) didn’t receive two or more doses of TT vaccination [9]. Worldwide, about 36% of mothers were not immunized with TT protective dose at birth against tetanus [10]. Consequently; about 49,000 neonates and a significant numbers of mothers died of tetanus [11]. This problem is much higher in South East Asian (SEA) and Sub-Saharan African (SSA) countries where 90% of maternal and neonatal tetanus (MNT) appeared and almost all cases ended with death [11]. In Ethiopia, about 51% of pregnant women were not received valid TT vaccination [12]. The problem magnified in Amhara regional state where 56% infants were not protected at birth(PAB) against tetanus [13].

To alleviate this problem, TT vaccination of pregnant women was included in World Health Organization’s (WHO) Expanded Program on Immunization (EPI) [14, 15]. World Health Organization (WHO) and its partners jointly have been working for global elimination of MNT basically through maternal TT vaccination. Accordingly, they had planned a targeted date for this goal, a reduction of neonatal tetanus incidence to below one case per 1000 live births per year in every district of the globe, to be at 1995. However, it was postponed many times due to inability to achieve the goal [14, 15]. In Ethiopia context, the Federal Ministry of Health (FMOH), in collaboration with Expanded Program of Immunization (EPI) partners, started implementing TT supplemental immunization since 1999 with the aim of MNT elimination. Moreover, the country introduced the protection at birth(PAB) method for tetanus toxoid (TT) immunization monitoring in 2009 [1]. By August 2015, 21 countries have still not reached the MNT elimination status including Somali region of Ethiopia [11].

Despite impressive efforts, coverage of PAB against tetanus is still low [9, 13–20]. Though, Ethiopia planned the national tetanus PAB coverage to reach 86% by the year 2015, it is still only 49% as of EDHS 2016 [12, 21]. Even, this magnitude is based mostly on health institutional data. Thus, this secondary data often may not depict the actual condition since the situation tends to happen in a communities with restricted access and/ or use of health facilities [22].

Therefore; with the current universal push of ending preventable deaths of newborns and children under 5 years of age, a reliable community based study on preventive strategies like TTPDI and associated factors to have representative data, is required. Having this point of view, this community based study was conducted to assess TTPDI and associated factors at last birth among mothers delivered within one year prior to the study in Debretabor town, North West Ethiopia, 2016.

## Methods

A community based cross sectional study was conducted at DebreTabor town which is the largest town in South Gondar zone Amhara regional state, Northwest Ethiopia. The town is located at 665 km from Addis Ababa (the capital city of Ethiopia) in Northwest direction.

Based on the 2007 National Census conducted by Central Statistical Agency of Ethiopia (CSAE), the projected total population in the town is about 55,596 of whom 27, 644 (49.7%) are men and 27,952 (50.3%) are women. Orthodox Christians’ are 96.72% of the population of the town and 2.54% are Muslims [23]. The town is divided in to four small administrative units called kebeles and has one general hospital, 3 health centers and 4 Health posts [23].

In the study area, antenatal health services are not active. In other words, if women did not attend the clinic, health providers don’t go to the door to give the care. However, the health extension workers (HEWs) are responsible to go to the community home –to- home to mobilize the pregnant women, their family and the community at large so as to visit the health facilities during pregnancy for antenatal care (ANC) service. In addition, HEWs provide information for the client on the importance of antenatal care follow up by moving door to door. As far as duties for provision of ANC services concerned, midwives are primarily responsible at the health centers as well as at the hospitals. Whereas, health extension workers are responsible at health post (i.e. the smallest unit of health facility). Furthermore, there is a consultancy system between midwives and other health care professionals.

Sample size for the study was determined using an Open-Epi Version 2 software by considering the following assumptions: ratio among married and unmarried women 1:1, OR 1.83, power 80%, CI 95%and the proportion of births PAB against tetanus among married women 77.9% [24]. So, the largest sample size for this study was 460 and by assuming 10% non response rate the final sample size turned to be 511.

All mothers in the town who gave birth within one year before the study period were included in the study. Mothers who came from other places to the town for the first time after delivery and also who resided in the town for less than six months were excluded.

First, total numbers of households (HHs), (13,837), were obtained from DebreTabor town administrative office. The sampling interval was made by dividing the total households (HHs) for total sample sizes by assuming every household (HH) had at least one eligible mother, (that is, 13,837/511 = 27). The first HH was then selected by using lottery method among the first 1–27 HHs. And then, selection was proceeded every 27 HHs interval by employing systematic random sampling technique. Finally, a total of 511 HHs with eligible mothers were selected. In case of no eligible woman in the HH, the interviewer approached to the next closest HH. In the selected HH, adequate explanation offered; presence of eligible mother confirmed; written informed consent requested and obtained; and then the HH identification code placed at questionnaire as well as at the door. Face to face interview was then proceeded by using structured and pretested questionnaire. At times, clients have been categorized as ever or never taking TT based on their verbal history. For those mothers who admitted ever history of receiving TT dose(s), documented evidences (either TT card or EPI registration book) was reviewed. Finally, mothers categorized as whether receiving tetanus toxoid protective dose immunization or not based on the listed criteria.

Tetanus toxoid protective dose immunization (TTPDI) was defined as proportion of mothers who have been received any of the following documented TT doses:

1. Two tetanus toxoid injections during that pregnancy
2. Two or more injections, the last one within 3 years of the birth
3. Three or more injections, the last one within 5 years of the birth
4. Four or more injections, the last one within 10 years of the birth
5. Five or more injections at any time prior to the birth

Socio-demographic, obstetric and health service related variables such as maternal age, mother’s educational status, husband’s educational status, marital status, religion, mother’s occupation, husband’s occupation, family monthly income, ethnicity, owing radio /television, parity, planned last pregnancy, having ANC visit during the recent pregnancy, number of ANC visit, time of initiating ANC visit, place of ANC visit, client perceived quality of services, time to cover the distance home to health facility, client perceived confidentiality, client trust on TT service, client perceived behavior of service provider, client perceived providers’ respect of clients, purpose of taking the vaccine and reason for not taking the vaccine. In addition, checklists on regarding TT vaccination status in the recent pregnancy as well as in life like ever taking the vaccination, number of doses, timing(interval) among doses, source of information for vaccination status which categorized as history alone, card(document) alone, or both history and card. Finally, TT protective dose immunization status (yes or no) was included.

The questionnaires were first prepared in English [Additional file 1] and translated into Amharic and finally, translated back to English. Six unemployed midwifery professional graduates were recruited for data collection process. These include, four diploma midwives for data collection and two BSc midwives for supervision. A one day training was given for data collectors and supervisors. In addition, data collectors had employed the interview guide during discussion [Additional file 2]. A pretest study was done on 5% of sample size. Data were checked, coded and entered to EPI INFO version 7. Then, it was exported to SPSS version 20 for analysis. Both descriptive and analytical statistical procedures were utilized. Descriptive statistics were carried out and the results were presented using tables and texts. Binary logistic regression was used to identify factors associated with TTPDI at last birth. Then, those variables with *P*-value < 0.2 from Bivariate analysis, were fitted to multivariable logistic regression with Backward Stepwise Likelihood Ratio. Both COR and AOR with the corresponding 95%CI were computed to show the strength of the association. Finally, statistically significant association of variables was determined based on Adjusted Odds ratio with its 95% confidence interval and *p*-value ≤0.05.

## Result

### Socio-demographic and economic characteristics

A total of 511 women were interviewed.

The median age of the participants was 27 years with (IQR =7). Majority, (83.8%), of the participants were in age group 20–34 years. More than half, (50.9%), of women were housewife by occupation and 99, (19.4%), women have never attended any formal education. Large segment, (77.5%), of respondents’ family earned more than 36.00 US dollar per month [Table 1].

**Table 1:** Socio-demographic and economic characteristics of women, Debre Tabor town, Northwest Ethiopia, 2016 (*n* = 511)

Variables	Frequency	Percentage
Age (years		
18–19	15	2.9
20–34	428	83.8
35–49	68	13.3
Mothers’ educational status		
Never attend formal education	99	19.4
Primary school	46	9
Secondary school and above	366	71.6
Mother’s occupation		
House wife	260	50.9
Government employee	120	23.5
Merchant	68	13.3
Student	53	10.4
Others^a^	10	2
Marital status		
Married	476	93.2
Divorced	28	5.5
Others^b^	7	1.4
Family monthly income(in US dollar)		
≤ 18	22	4.3
18.01–36.00	93	18.2
≥ 18.01	396	77.5
Religion		
Orthodox	476	93.1
Muslim	33	6.5
Catholic	2	0.4
Radio /Television		
Yes	475	93.0
No	36	7.0
Husbands’ occupation		
Governmental employee	320	62.6
Merchant	69	23.5
Student	55	10.8
Farmer	43	8.4
Daily laborer	24	4.7
Husband’s education		
Never attend formal education	14	2.7
Primary school	92	18
Secondary school and above	405	79.3

^a^Daily labored and house worker

^b^Windowed and never married

### Obstetric and health service related factors

More than half, (52.8%), and 226, (44.2%), of the participants were primipara and grand-multipara, respectively. Considerable, (90%), of participants’ last pregnancy were planned. Substantial proportion, (95.3%), of the respondents have had ANC visit with the mean ± SD of 2.14 ± 1.01 visits and about 36.6% of them have had 4 or more ANC visits. More than two-third, (74.2%), of women perceived as good quality of health services and 472, (92.4%), thought as the service provider respected the women. Majority, (86.1%), women believed as there was no confidentiality problem while about 480, (93.9%), of them trusted health care providers [Table 2].

**Table 2:** Obstetric and health service related factors among mothers in DebreTabor town, Northwest Ethiopia, 2016 (*n* = 511)

Variables	Frequencies	Percentage
Parity		
1	270	52.8
2–4	15	2.9
> 5	226	44.2
ANC visit		
Yes	487	95.3
No	24	4.7
Time of starting ANC visit (*n* = 487)		
< 13 wks	112	23
13–28 wks	256	52.6
> 28wks	119	24.4
Number of ANC visit (*n* = 487)		
One	38	7.8
Two	90	18.5
Three	172	35.3
Four or more	187	38.4
Site of ANC visit (*n* = 487)		
Health post	10	2.1
Health center	223	45.8
Hospital	254	52.2
Client perceived Quality of TT service		
Good	379	74.2
Poor	132	25.8
Client perceived behaviour of service providers		
Good	434	84.9
Poor	77	15.1
Time to cover the distance		
Less than 1 h	350	68.5
Between 1 and 2 h	134	26.2
More than 2 h	27	5.3
Ever received TT		
Yes	477	93.3
No	34	6.7
Number of TT vaccination (*n* = 477)		
One	100	20.96
Two	242	50.73
Three	68	14.26
Four	53	11.11
Five or above	14	2.94
Purpose of taking TT vaccination (*n* = 477)		
For the mother	28	5.9
For the newborn	43	9.0
For both	393	82.4
Not known	13	2.7

### Proportion of tetanus toxoid protective dose immunization

Proportion of mothers who have been immunized with TT protective dose immunization were 56.2% (95%CI: 52–60%). Among mothers who did not take TT immunization properly; majority, (53.26%), replied that no one advised them and others reasons were not known (30.43%), no problem faced (11.96%), distance barrier (3.26%) and no service provider (1.09%).

### Factors influencing tetanus toxoid protective dose immunization

#### Bivariate analysis

Finding from bivariate logistic regression analysis showed that mother’s age, mother’s educational status, marital status, ever having ANC visit, parity, perceived behavior of service providers, place of ANC visit, time of initiating ANC visit, planned last pregnancy, number of ANC visit and perceived quality of service were associated with TTPDI at last birth **[**Table 4]. Whereas, among socio-demographic variables, only mothers’ age and educational status were stasticaly associated with ever taking TT vaccination **[**Table 3**]**.

**Table 3:** Comparisons of socio-demographic variables across TT ever and never vaccinated women in Debretabor town, Northwest Ethiopia, 2016

Socio demographic Variables	Ever taking TT Vaccine?		COR with 95% CI	AOR with 95% CI
	Yes n (%)	No n (%)		
Mothers’ age (in years)				
18–29	349 (91.6)	32 (8.4)	1	1
30–49	128 (98.5)	2 (1.5%)	5.87 (1.39,24.84)*	7.27 (1.70,31.15)*
Mothers’ religious				
Orthodox Tewahido	445 (93.5)	31 (6.5)	1.35 (0.39,4.64)	1.17 (0.29,4.67)
Others^a^	32 (91.4)	3 (8.6)	1	1
Mothers’ educational status				
Never attend formal education	87 (87.9)	12 (12.1)	1	1
Attend formal education	390 (94.7)	22 (5.3)	2.45 (1.17,5.13)*	4.02 (1.69,9.58)*
Mother’s occupation				
House wife	244 (93.8)	16 (6.2)	1.18 (0.59,2.37)	2.13 (0.94,4.81)
Others^b^	233 (92.8)	18 (7.2)	1	1
Marital status				
Currently Married	446 (93.7)	30 (6.3)	1.92 (0.64,5.79)	2.15 (0.67,6.98)
Currently not married	31 (88.6)	4 (11.4)	1	1
Family monthly income(in US dollar)				
≤ 36	108 (93.9	7 (6.1)	1	1
> 36	369 (93.2)	27 (6.8	0.89 (0.38,2.09)	0.57 (0.07,5.04)
Have Radio /Television				
Yes	442 (93.1)	33 (6.9)	0.38 (0.05,2.88)	0.28 (0.04,2.21)
No	42 (97.2)	1 (2.8)	1	1
Husbands’ occupation				
Governmental employee	300 (93.8)	20 (6.2)	1.19 (0.59,2.41)	1.14 (0.52,2.51)
Others^c^	177 (92.7)	14 (7.3)	1	1
Husband’s education				
Never attend formal education	463 (93.2)	34 (6.8)	NA	NA
Attend formal education	14 (100)	0		

*P-value < 0.05

^a^Catholic and protestant

^b^ Daily labored, house worker, merchant and student

^c^ Student, merchant, farmer and daily labored

#### Multivariable analysis

In the multivariable logistic regression analysis; mother’s educational status, planned pregnancy, perceived quality of service, time of initiation and number of ANC visit remained statistically significantly associated with TTPDI.

Accordingly, mothers with formal education were 2.09 times more likely to have been immunized with protective doses of TT than uneducated mothers (AOR = 2.09; 95 CI: 1.12, 3.90).

Besides, the odds of having TT protective dose immunization were nearly 4 times higher among mothers who have initiated ANC visits with in first trimester compared to their counterparts (AOR = 4.29; 95%CI: 1.94,9.49). Similarly, mothers who had four or more ANC visits during the last pregnancy were 5.16 times more likely to have been immunized with TT protective dose than those who had below four ANC visit (AOR = 5.16; 95%CI: 2.93, 11.14). This study also depicted that mothers with planned last pregnancy were 6.63 times more probably to receive TT protective dose immunization than their counterpart (AOR = 6.63; 95% CI: 2.36, 18.63).

Finally, Mothers, having good perceived quality of service were 2.20 times more probably to have been immunized with protective doses of TT than their congruent (AOR = 2.20; 95% CI:1.26, 3.84) [Table 4].

**Table 4:** Factors influencing tetanus toxoid protective dose immunization among mothers, DebreTabor town, Northwest Ethiopia, 2016 (*n* = 511)

Variables	TT Protective dose		Crude Odds Ratio 95% C1	*P*-Value	Adjusted Odds Ratio 95% C1
	Yes n (%)	No n (%)			
Age					
18–29	203 (53.3)	178 (46.7)	0.63 (0.41,0.94)	0.02	0.59 (0.33,1.08)
30–49	84 (64.6)	46 (35.4)	1		1
Religion					
Orthodox	271 (56.9)	205 (43.1)	1.57 (0.32,1.27)	0.2	NA
Others^a^	16 (45.7)	19 (4.3)	1		
Educational status					
Ever attended	249 (60.4)	163 (39.6)	2.45 (1.56, 3.890	0.000	2.09 (1.12,3.90)*
Never attended	38 (38.4)	61 (61.6)	1		1
Marital status					
In marital relation	277 (58.2)	199 (41.8)	3.48 (1.64,7.41)	0.002	1.58 (0.50,4.98)
No marital relation	10 (28.6)	25 (71.4)	1		1
Husband educational status					
Ever attended	282 (56.7)	215 (43.3)	2.36 (0.78–7.15)	0.16	1.16 (0.19,7.07)
Never attended	5 (35.7)	9 (64.3)	1		1
Mother’s occupation					
House wife	145 (55.8)	115 (44.2)	0.97 (0.68,1.37)	0.86	NA
Others^b^	142 (56.6)	109 (43.4)	1		
Husband’s occupation					
Governmental employee	182 (56.9)	138 (43.1)	1.08 (0.75,1.55)	0.68	NA
Others^c^	105 (55)	86 (45)	1		
Owing radio/Television					
Yes	263 (55.4)	212 (44.6)	0.62 (0.30,1.27)	0.20	NA
No	24 (66.7)	12 (33.3)	1		
Planned last pregnancy					
Yes	278 (60.4)	182 (39.6)	7.13 (3.39–15.00)	0.000	6.63 (2.36,18.63)*
No	9 (17.6)	42 (82.4)	1		1
Time to cover distance					
< 1 h	179 (51.14)	171 (48.86)	1.03 (0.47,5.38)	0.36	NA
> or = 1 h	81 (50.31)	80 (49.69)	1		
Family monthly income(in US dollar)					
< or = 36	57 (49.6)	58 (50.4)	1		
> 36	230 (58.1)	166 (41.9)	1.41 (0.93,2.14)	0.20	NA
Ever having ANC visit					
Yes	289 (58.7)	201 (41.3)	3.49 (2.28,4.86)	0.000	1.48 (0.70,3.09)
No	7 (29.17)	17 (70.83)	1		1
Place of ANC visit in recent pregnancy(n = 487)					
Hospital	132 (51.97)	122 (48.03)	1.00 (0.36,3.72)	0.53	NA
Health Center/Post	121 (51.93)	112 (48.07)	1		
Timing of initiating ANC visit(n = 487)					
Within 1st trimester	101 (90.2)	11 (9.8)	9.43 (4.9,18.14)	0.000	4.29 (1.94,9.49)**
After 1st trimester	185 (49.3)	190 (50.7)	1		1
Parity					
Two or more	113 (46.9)	128 (53.1)	0.49 (0.336,0.684)	0.000	0.94 (0.48,1.85)
One	174 (64.4)	96 (35.6)	1		
Number of ANC visit (n = 487)					
Four or above	151 (80.7)1	36 (19.3)	5.13 (3.34,7.87)	0.000	5.16 (2.93,11.14)
Three or below	135 (45)	165 (55)	1		1
Client perceived quality of service					
Good	249 (65.7)	130 (34.2)	4.74 (3.08,7.30)	0.000	2.20 (1.26,3.84)*
Poor	38 (28.8)	94 (71.2)	1		1
Client perceived behavior of service provider					
Good	270 (62.2)	164 (37.8)	5.81 (3.28,10.30)	0.000	1.84 (0.84,4.05)
Poor	17 (22.1)	60 (77.9)	1		1

*P-value < 0.05

^a^Catholic and protestant

^b^Daily labored, house worker, merchant and student

^c^Student, merchant, farmer and daily labored

## Discussion

This study assessed proportion of tetanus toxoid protective dose immunization and associated factors among mothers who gave birth within one year prior to data collection period. The odds of TT protective dose immunization was higher among mothers who have attended formal education, have initiated early ANC visit, with planned last pregnancy, achieving two or more ANC visit, and with perceived good quality of service.

The study illustrated that the proportion of Tetanus toxoid protective dose immunization among mothers found to be 56.2%. The finding is in line with the Nigeria national coverage (55%) [25]. On the other hand, this magnitude is higher as compared to the study done in Lagos Nigeria (20%) [26], Umuahia Nigeria (50%) [27], Ankara Turk (27.8%) [28] and Ibadan Nigeria (38.1%) [29].

This result was also larger as compared to the Ethiopian national PAB coverage (49%) [12] and other local reports, such as Ambo town,(26.8%) [24], Afar regional (26.7%), [16] and Amhara regional PAB coverage, (43.2%), [16].

This disparity could be explained by variation in tools used to measure the outcome variable: some studies were laboratory based which measured the actual serum antibody level enough to protect against tetanus where as the current study used documented evidence to measure proportion of TT protective dose. So, the tool used in the current study might brought about higher magnitude as compared to that of the actual serum antibody measurement [29]. Besides, the discrepancy could be due to variation in the study setting; some studies have included both urban and rural residents. Studies showed that rural dwellers were less educated as compared to urban women. As the current and other studies showed, TT vaccination utilization predicted by level of mother’s education (13). So, the higher magnitude of TT protective dose immunization in the current studies might be attributable to composition of the study participants that is including only urban women. In addition, as studies elucidated, lack of TT vaccination occurred in the communities with limited access to and use of healthcare services, and maternal TT vaccination was predicted by distance from the nearest immunization centre, and level of contact with health worker (35, 38, 45). Thus; those women resided in the rural and remote areas would not easily accessed to health facilities as compared to urban women although the TT service was free of charge [24]. Furthermore, the discrepancy could be variation in time of the study: some studies were done back to 5 years. But, currently trends on educational status and awareness toward the importance of TT changed across a time. These all mentioned explanation possibly could answer why TT protective dose immunization in the current study was higher.

However, the result was considerably lower as compared to findings from national PAB coverage in Ghana (82.6%) [30] and local studies in: Pakistan in 2007-(87%)-[31], Pakistan in 2011-(90.5%)-[32], Ghana (82.6%) [30], Sudan (83%) [33] and Ethiopia (82.3%) [13]. This variation might be due to the method employed to measure the outcome variable; the current study used proofed (documented) evidence for maternal vaccination status whereas most other studies described primarily based on maternal verbal history which was prone to recalled bias and might be resulted in over estimation in the latter studies [30–33].

As this study demonstrated, the odd of TT protective dose immunization was 2.09 times among mothers with formal education than that of uneducated women. This finding is in line with the study done in different settings: in Bangladesh [34], in France [35] and in Ethiopia [24, 36]. This might be due to the fact that education increases awareness of women on the benefits of immunization, a greater decision-making power at home and perhaps escalate capacity to travel outside the home to seek health care (26).

This study also revealed that planned last pregnancy was statistically significantly associated with TT protective dose immunization; women with planned last pregnancy were more than six times more likely to have been immunized with TT protective dose than women whose pregnancy was unplanned. This is in accordance with the study done in Bangladesh [37], Kenya [38] and Ambo town,Ethiopia [24]. This might be due to the logic those mothers with planned pregnancy possibly ready themselves to overcome physical barriers like distance, to allocate time to seek health care, to attend health related media promotion like ‘the first 1000 days’ and probably to search health related information either, by consulting health care providers or by reading related papers.

According to this study, TT protective dose immunization was independently predicted by frequency of ANC visit during the recent pregnancy. Hence, mothers who had four or more number of ANC visit during the last pregnancy were more than five folds more likely to have been immunized with protective dose of TT than their counter parts. This implies that attending the recommended number of ANC visit is a good opportunity for valid TT immunization utilization. This was with regard to the study done in Peshawar, Pakistan [39] and in Kenya [38]. This could be due to the reality that the higher the frequency the mother visited health facilities, the higher the mothers received appropriate health care services and correspondingly, the valid the TT doses offered. Plus to that, as the contact time with health professional increased, the mother got more health related information and this in turn might change her health seeking behavior positively.

This study also elucidated that TT protective dose immunization was influenced by time of initiating ANC visit during the last pregnancy. Mothers with history of initiating ANC visit within the first trimester were nearly four folds more likely to have been immunized with this dose than those starting later. This suggests that if mothers started ANC visit in early gestation, they would get adequate time enough to take two or more TT vaccination based on the schedule prior to giving birth. This finding is in agreement with the study done in Lahore district, Pakistan [31].

Based on the current study, the odds of TT protective dose immunization was 2.20 times higher among mothers with perceived good quality of service than their counter parts. This suggested that no matter the real quality of service provided, simply client perception toward quality of service, matters receiving valid TT vaccination. This is consistent with the study conducted in Dormaa, Ghana [40]. This result also supported by study conducted in Hispanic and Houston, Texas [41].

## Limitation of the study

The study was conducted solely in the urban area. As a result; it did not involve participants from rural areas. In addition, although using documented evidence for confirming protective dose immunization could be taken as strength, the finding of this study might be underestimated as compared to other studies which reported based on maternal verbal history alone.

## Conclusion

The proportion of mothers who have been vaccinated with protective doses of TT immunization in the study area was below the national target. Formal education, planned last pregnancy, timely ANC visit, four or more ANC visit and perceived good quality of service were factors positively associated with protective doses of TT immunization. Thus; complete ANC visit, planned pregnancy and early initiation of ANC visit should be emphasized. Similarly; female education and correcting women perception toward quality of TT service is recommended.

## Additional files

Additional file 1: English language copy of the questionnaire. English version of Questionnaire for the study conducted on tetanus toxoid protective dose immunization and associated factors among mothers who gave birth within one year prior to the study in Debre Tabor Town, Northwest Ethiopia, 2016. (DOCX 24 kb)

Additional file 2: Interview Guide. Interview guide during the discussion with participants for the study conducted on tetanus toxoid protective dose immunization and associated factors among mothers who gave birth within one year prior to the study in Debre Tabor Town, Northwest Ethiopia, 2016. (DOCX 23 kb)

Additional file 3: Study participant's consent sheet. English version of consent sheet for the study conducted on tetanus toxoid protective dose immunization and associated factors among mothers who gave birth within one year prior to the study in Debre Tabor Town, Northwest Ethiopia, 2016. (DOCX 14 kb)

## Abbreviations

ANC: Ante Natal Care
AOR: Adjusted Odds Ratio
COR: Crude Odds Ratio
EDHS: Ethiopian Demography and Health Survey
EPI: Expanded Program on Immunization
FMOH: Federal Ministry of Health
HEWs: Health Extension Workers
HH: House Hold
MNT: Maternal and Neonatal Tetanus
NNT: Neo Natal Tetanus
PAB: Protected at Birth
SPSS: Statistical Package for Social Science
TT: Tetanus Toxoid
TTTPDI: Tetanus Toxoid Protective Dose Immunization
WHO: World Health Organization

## References

[1] Minisitry of Health (2010). Ethiopia national Expanded Programme on Immunization.

[2] Federal Republic of Ethiopia Minstry of Health (2007). HMIS / M&E Indicator Definitions HMIS / M&E Redesign: Technical Standards: Area 1 version 1.0.

[3] World Health Organization (2006). Tetanus vaccine. Weekly epidemiological record.

[4] Thwaites CL, Farrar JJ (2003). Preventing and treating tetanus -The challenge continues in the face of neglect and lack of research. Biomed J.

[5] World Health Organization (2015). Protection at birth (PAB) against tetanus. Global Health Observatory data.

[6] World Health Organization. Expanded Programme on immunization Ethiopia. Ethiopia Country Office; 2014. http://www.who.int/countries/eth/areas/immunization/en/.

[7] Blencowe H, Lawn J, Vandelaer J, Roper M, Cousens S. Tetanus toxoid immunization to reduce mortality from neonatal tetanus. Int J Epidemol. 2010;27:102–9. 10.1093/ije/dyq027 PMC2845866 20348112

[8] Demicheli V, Barale A, Rivetti A. Vaccines for women for preventing neonatal tetanus. Cochrane Collaboration. Cochrane Database Syst Rev. 2015:1. 10.1002/14651858.CD002959.pub4 PMC7138051 26144877

[9] Thwaites CL, Loan HT (2015). Eradication of tetanus. Oxford J British Med Bulletin.

[10] World Health Organization. Neonatal tetanus (PAB)data by WHO region: Global health observatory data repository; 2015. http://www.who.int/gho/immunization/neonatal_tetanus/en/.

[11] World Health Organization. Maternal and neonatal tetanus (MNT) elimination-the initiative and challenges. Immun Vaccin Biol. 2015;21:1–2.

[12] Central Statistical A (2016). Ethiopian demographic and health survey key indicators report.

[13] Ethiopia Central Statistical Agency (2012). Ethiopia Demographic and Health Survey 2011.

[14] World Health Organization: Weekly epidemiological record. 2007;82:141–52. http://www.who.int/wer/2007/en/.

[15] Verma R, Khanna P. Tetanus toxoid vaccine: elimination of neonatal tetanus in selected states of India: Human vaccines & immunotherapeutics; 2012. https://www.ncbi.nlm.nih.gov/pmc/articles/PMC3660763/. 10.4161/hv.21145 PMC3660763 22894950

[16] World Health Organization. neonatal tetanus. Immun Vaccin Biol. 2015;512:1–4.

[17] Khan R, Vandelaer J, Yakubu A, Raza AA, Zulu F (2015). Maternal and neonatal tetanus elimination: from protecting women and newborns to protecting all. J Women's Health.

[18] The Federal Democratic Republic of Ethiopia Ministry of Health (2015). Health Sector Transformation Plan.

[19] Kidane T, Yigzaw A, Sahilemariam Y, Bulto T, Mengistu H, Belay T, Bisrat F, Benti D, Mbakuliyemo N, Olusegum B. National EPI coverage survey report in Ethiopia, 2006. Ethiop J Health Dev. 2008;22:1–5.

[20] WHO and UNICEF (2014). Estimates of immunization coverage.

[21] Federal Democratic Republic of Ethiopia Ministry of Health (2014). Health Sector Development Programme IV.

[22] Ethiopia Central Statistical Agency (2005). Tetanus Toxoid Vaccination.

[23] Federal Democratic Republic of Ethiopia Population Census Commission (2008). Summary and statistical report of the 2007 population and housing census :population size by age and sex. United Nations Population Fund.

[24] Adugna E (2011). factors influencing tetanus toxoid immunization and protection at birth coverage among child breang age women of Ambo town and its surrounding area.

[25] World Health Organization (2015). Protection at birth against neonatal tetanus(PAB) data by WHO Region.

[26] Sule SS, Nkem-Uchendu C, Onajole AT, Ogunowo BE (2014). Awareness, perception and coverage of tetanus immunisation in women of child bearing age in an urban district of Lagos, Nigeria. Niger Postgraduate Med J.

[27] Nwokeukwu HI, Ukegbu AU, UE-U KCN, Nwankwo N, Osunkwo D, Ajuogu E (2014). Tetanus Toxoid Immunization Coverage in Federal Medical Centre, Umuahia, Abia State,South East Zone, Nigeria. International journal of tropical disease & health.

[28] Maral I, Baykan Z, Aksakal FN, Kayikcioglu F, Bumin MA (2011). Tetanus immunization in pregnant women: evaluation of maternal tetanus vaccination status and factors affecting rate of vaccination coverage. Public Health.

[29] Orimadegun AE (2014). Akinlolu a Adepoju, Olusegun O Akinyinka: prevalence and socio-demographic factors associated with non-protective immunity against tetanus among high school adolescents girls in Nigeria. Ital J Pediatr.

[30] Diamenu SK, Bosnu G, Abotsi F, Tweneboa PO, Okoh-Owusu M, Amoh P, Yakubu A, Khan R (2015). Introducing protection at birth (Pab) method of monitoring tetanus-diphtheria (td) vaccination coverage of mothers in Ghana. International Journal of Vaccines and Immunization.

[31] Hasnain S, Sheikh NH (2007). Causes of low tetanus toxoid vacccinatiuon coverage in pregnant women in Lahore District,Pakistan. East Mediterr Health J.

[32] Hashmi FK, Islam M, Khan TA, Tipu MK. Vaccination coverage of mothers during pregnancy with tetanus toxoid and infants after Birth. Pakistan J Pharmacy. 2011;24(2):1–3.

[33] Awad H, Sultan S (2009). Assessment of tetanus toxoid coverage and protection at birth against neonatal tetanus in Khartoum state, Sudan.

[34] Rahman M. Determinants of the utilization of the tetanus toxoid (TT) vaccination coverage in Bangladesh: evidence from a Bangladesh demographic health survey 2010. Internet J Health. 2010;8(2):3–5.

[35] Guthmann JP, Fonteneau L, Antona D, Lévy-Bruhl D (2010). Factors associated with tetanus vaccination coverage in adults in France and with knowledge of vaccination status. Medecine et maladies infectieuses.

[36] Walle F, Kassa M. Coverage and factors associated with tetanus toxoid vaccination among private college students, Bahirdar, Ethiopia. J Vaccine. 2013;4:106.

[37] Rahman M (2009). Tetanus toxoid vaccination coverage and differential between urban and rural areas of Bangladesh. East African journal of public health.

[38] Zelalem TH, Chertok IRA, Teweldeberhan AK (2013). Determinants of utilization of sufficient tetanus toxoid immunization during pregnancy: evidence from the Kenya demographic and health survey, 2008–2009. J Community Health.

[39] Naeem M, Khan MZ-U-I, Abbas SH, Adil M, Khan A, Naz SM, Khan MU. coverage and factors associated with tetanus toxoid vaccination among married women of reproductive age: a cross sectional study in Peshawar. J Ayub Med College. 2010;22(3):1. 22338439

[40] Anokye M, John A, Mensah, Frank O, Frimpong, Emelia O, Aboagye, Acheampong N (2014). Immunization coverage of pregnant women with tetanus toxoid vaccine in Dormaa East District-Brong Adaro region, Ghana. Mathematical Theory and Modeling.

[41] Beel ER, Rench MA, Montesinos DP, Mayes B, Mary Healy C (2013). Knowledge and attitudes of postpartum women toward immunization during pregnancy and the peripartum period. Human vaccines & immunotherapeutics.

