# Supplementary material for: The role of timely initiation of antenatal care on protective dose tetanus toxoid immunization: the case of northern Ethiopia post natal mothers
Source: BMC Pregnancy Childbirth. 2018 Jun 15;18:235. doi: 10.1186/s12884-018-1878-y (PMC6003212; doi:10.1186/s12884-018-1878-y)
Supplement: Supplementary file 1 — English language copy of the questionnaire. English version of Questionnaire for the study conducted on tetanus toxoid protective dose immunization and associated factors among mothers who gave birth within one year prior to the study in Debre Tabor Town, Northwest Ethiopia, 2016. (DOCX 24 kb) [file 12884_2018_1878_MOESM1_ESM.docx]

Additional file 1: English version Questionnaire for community based survey on Tetanus toxoid protection at birth and associated factors among mothers who gave birth within the last one year, in Debre Tabor Town Northwest Ethiopia, 2016

Households Identification

001. Questionnaire Code___________

002. Kebele __________

003. How long have been living in this town/Area ------

004. Time table for visiting/revising a household

|  | First visit | Second visit | Third visit |
| --- | --- | --- | --- |
| Date |  |  |  |
| Interviewer |  |  |  |
| Result |  |  |  |

Results of visiting:

1. Completed, 2.Rejected, 3.No response, 4.Partial response 5. Other

• Show the answer of the respondent by circling the number and / or writing on the space provided.

• Time of interview start__________ ended_____________

English version of the questionnaire

Part I -Questionnaire on socio-demographic characteristics.

| No | Questionnaire | Alternative choice for responses | Skip to code |
| --- | --- | --- | --- |
| 101 | What is your age(in year) | _______ |  |
| 102 | What is the highest level of  schooling you have attended | 1. Unable to read and write  2. Read and write with no formal education  3. Primary school (grade 1-8)  4. Secondary/high school grade (9-10)  5.Preparatory school( grade 11-12)  6. university or college  99. Other Specify |  |
| 103 | What ethnic group do you  Belong? | 1. Amhara  2.Tigrie  3. Oromo  4. Guragie  99. Other specify_____________ |  |
| 104 | What is your occupation? | 1. Housewife  2. House worker/maid servant)  3. Civil servant  4. Merchant  5. Student  6. Daily laborer  99. Other specify |  |
| 105 | What is your religion? | 1. Orthodox Tewahido  2. Catholic  3. Protestant  4. Muslim  99. Other specify |  |
| 106 | What is your marital status? | 1. Married  2. Divorced  3. Widowed  4. unmarried  99.Otrher specify |  |
| 107 | What is your family Monthly income (in Ethiopian Birr)? | _______________ |  |
| 108 | Husbands’ Occupation | 1. Governmental Employee  2. Farmer  3. Daily laborer  4. Merchant  5. Student  99. Other specify |  |
| 109 | Do you have radio/Television | Radio- 1. Yes 2. No  Television- 1. Yes 2. No |  |
| 111 | Husband Educational status | 1. Unable to read and write  2. Read and write with no formal education  3. Primary school(Grade 1-8)  4. Secondary school( grade 9-10)  5. Preparatory school(grade 11-12)  6. University or college  99. Other Specify |  |

Part II: Questionnaire on Obstetric determinants

| Code | Questionnaire | Alternative | Skip to code |
| --- | --- | --- | --- |
| 201 | Parity | ____________ |  |
| 202 | Planned last pregnancy? | 1. Yes 2. No |  |
| 203 | Did you have ANC visits in the last pregnancy | 1. Yes 2. No |  |
| 204 | When did you start ANC visit in the last pregnancy(gestational age by week) | ----------------- |  |
| 205 | Place of ANC visit? | 1. Health Post  2. Health Centre  3. Hospital  4. Private clinic  99.if any other specify |  |
| 206 | What was the total number of ANC visits during the index pregnancy? | ------------- |  |

Part III: Questionnaire on TT immunization

| 301 | During your index pregnancy, were  you given TT injection? | 1. yes  2. No  3. Don’t remember |  |
| --- | --- | --- | --- |
| 302. | -If yes for question number 301, how many TT doses did you take? | ------------dose(s) |  |
| 303. | Time(interval) of TT dose(s)during last pregnancy(if she had given) | 1.B/nTT1&TT2(if any) ---months  2.B/n TT2&TT3(if any)----months  3.B/n TT3&TT4(if any)---months  4.B/n TT4&TT5(if any) ----months |  |
| 304 | Place of TT dose given | 1. Health Post  2. Health Centre  3. Hospital  4. Home/Out reach  99.if any other specify |  |
| 305 | Immunization status(Dose)  Total(including during the last pregnancy or prior to the index pregnancy | 1. None vaccinated  2. TT1  3. TT2  4. TT3  5. TT4  6. TT5  7. Unknown |  |
| 306 | TT doses interval (regarding total TT doses intervals including before or during the last pregnancy | 1.B/nTT1&TT2(if) any ---months  2.B/n TT2&TT3(if any)----months  3.B/n TT3&TT4(if any)---months  4.B/n TT4&TT5(if any) ----months |  |
| 307 | Date of each dose? | TT1___________  TT2______________  TT3_________________  TT4______________  TT5_________________ |  |
| 308 | Purpose of getting injection | 1. To prevent self from Tetanus  2. To prevent Child from Tetanus  3. To prevent both, self and child  from tetanus  4. Don’t know  99. other specify |  |
| 309 | Reason not getting TT injection  (more than one answer is possible) | 1. Not aware  2. Nobody advice  3 Service area too far  4 Fear of side effects  4. Provider not available  5. No problem experienced  99. Other specify |  |
| 310 | Source of information for TT vaccination | 1. History alone  2. Card alone  3. Both |  |
| 311 | Did the mother received TTPDI during the index birth | 1. Yes 2. No |  |

Part IV: Question on respondents view on quality of immunization services

| No | Questionnaire on identification of the respondents | Alternative choices for responses | Skip to Code |
| --- | --- | --- | --- |
| 401 | Were the health workers respectful? | 1. Yes  2. No  99. Don’t know |  |
| 402 | What is your feeling about the quality of Services given? | 1. Good  2. Poor  99. Don’t know |  |
| 403 | Do you trust on the service provided at that health institution? | 1. Yes  2. No  99. Don’t know |  |
| 404 | How was the behavior of health Workers providing immunization services? | 1.Good  2. Poor  99. Don’t know |  |
| 405 | How long does it take to travel from your home to the nearest health institution (in Minute? | ---------------- |  |

“Thank you very much for your cooperation!”
