# Supplementary material for: The role of timely initiation of antenatal care on protective dose tetanus toxoid immunization: the case of northern Ethiopia post natal mothers
Source: BMC Pregnancy Childbirth. 2018 Jun 15;18:235. doi: 10.1186/s12884-018-1878-y (PMC6003212; doi:10.1186/s12884-018-1878-y)
Supplement: Supplementary file 2 — Interview Guide. Interview guide during the discussion with participants for the study conducted on tetanus toxoid protective dose immunization and associated factors among mothers who gave birth within one year prior to the study in Debre Tabor Town, Northwest Ethiopia, 2016. (DOCX 23 kb) [file 12884_2018_1878_MOESM2_ESM.docx]

Additional file 2: Interview guide during discussion with participants for the study conducted on tetanus toxoid protective dose immunization and associated factors among mothers who gave birth who gave birth within one year prior to the study in Debre Tabor Town, Northwest Ethiopia, 2016

1. Select the household. Regarding selection of house hold:

- Select one initial household by bottle method;
- Select the first household by lottery method among the first 27 house holds
- If eligible mother avail on the selected household, conduct interview(follow interview guide number 2 and above) and then continue every 27 households
- If no eligible mother on the selected house hold, approach to the closest house hold and so forth.

1. Great politely and introduce yourself
2. Explain the purpose of the interview and assure for her confidentiality and privacy issue
3. Ask permission for interview
4. Check whether she is eligible or not. The mother is considered as eligible if she fulfilled the following criteria:

- If she had gave birth with in the first 1 year AND
- If she resided in the Debretabore town at least for six months AND
- If she come to Debretabore town at least 4 weeks prior to the index child birth

6. If the mother is volunteer but the current time is inconvenient for her:

- Request her convenient time and appoint on her preferable time
- If you have started interviewing and then discontinued due to different reasons and if she is still volunteer to continue on other time, thank her and appoint on her convenient time.

7. If she refused the interview, say thank you and good bye and then approach to the next nearest household

8. If she volunteered for interview, obtain signature, put the code both at the questionnaire and at the door, proceed interviewing by using face to face interview by keeping her privacy and confidentiality

9. While asking the monthly income;

- For farmers and others who have no fixed monthly income, first calculate the annual income and divided it for 12 which yields monthly income.
- Calculate all sources of family income ; not necessarily only the mother’s or the husband’s income

10. During asking the time initiation of ANC visit,

- Calculate the gestational age(GA) when she started ANC visit starting from her Last Normal Menstrual Period (LNMP)
- If the client didn’t remember the exact time, help her by showing directions(hints) for recalling like Holidays, and other events
- If the client did not remember the exact LNMP, use other possible methods of estimating GA like U/S, quickening, her verbal report of GA (that told by her health care provider at time of her first ANC visit)
- The client may report the time (Gestational Age) usually by months so change it to weeks

11. Regarding interviewing on TT, ask her whether she ever received TT vaccination or not verbally.

- If she denied receiving TT, skip variables concerning TT vaccination.
- If she admitted receiving TT, request her TT card for evidence. If she had lost her TT card, obtain medical registration number which used for searching her TT vaccination related data on EPI registration book at the health facility where she received the TT.
- After once obtained evidence for taking TT vaccination (either TT card or EPI registration book), fill the blank space provided at the check list section in the questionnaire like
- Total number of TT vaccination
- Interval of doses among total number of TT doses
- Number of TT vaccination during the index pregnancy
- Interval of doses among TT doses given the index pregnancy
- Date of each dose given
- Place of TT vaccination given

12. Then categorized mothers as receiving tetanus toxoid protective dose immunization or not based on the following criteria. Classify mothers as receiving protective doses of TT vaccination if she received any of the following documented TT doses:

1. Two tetanus toxoid injections during that pregnancy
2. Two or more injections, the last one within 3 years of the birth
3. Three or more injections, the last one within 5 years of the birth
4. Four or more injections, the last one within 10 years of the birth
5. Five or more injections at any time prior to the birth

13. After making sure that all variable are filled, leave the mother by acknowledging for her time and permission.
