# Supplementary material for: The role of timely initiation of antenatal care on protective dose tetanus toxoid immunization: the case of northern Ethiopia post natal mothers
Source: BMC Pregnancy Childbirth. 2018 Jun 15;18:235. doi: 10.1186/s12884-018-1878-y (PMC6003212; doi:10.1186/s12884-018-1878-y)
Supplement: Supplementary file 3 — Study participant's consent sheet. English version of consent sheet for the study conducted on tetanus toxoid protective dose immunization and associated factors among mothers who gave birth within one year prior to the study in Debre Tabor Town, Northwest Ethiopia, 2016. (DOCX 14 kb) [file 12884_2018_1878_MOESM3_ESM.docx]

Additional file 3: English version of participant’s consent sheet for the study conducted on tetanus toxoid protective dose immunization and associated factors among mothers who gave birth who gave birth within one year prior to the study in Debre Tabor Town, Northwest Ethiopia, 2016

Read the consent as it is!

Greetings Hello! My name is _________________ I am a team member involved in the study conducted by staff of university of Gondar ,college of medicine and health science department of midwifery on tetanus toxoid protective dose immunization and associated factors among mothers who gave birth who gave birth within the last one year ,in Debre Tabor Town North west Ethiopia ,2016

I am conducting a study on maternal Tetanus toxoid protective dose immunization coverage in DebreTabor Town.

The interview will take about 30 minutes. No information concerning you, as individual will not be passed to another individual or institution without your agreement.

You are kindly invited to be included in the study, which will have importance in improving maternal and child health services.

All information which you are being, asked to provide in this questionnaire will be kept strictly confidential. And, will be used only for study purposes.

Your participation is voluntary and you have the right to participate or not has been communicated. However, your participation is important to full fill the study purpose.

Contact Address

1. Principal investigator, Cell phone 0918473798, e-mail: muhabawshumye@gmail.com

2. Institutional review board -

Do you agree to answer the following questions to the best of your ability? Yes___No____

If your answers yes, please put your signature. Signature ----------------------------------

Please continue responding to the interview. And if no, thank and stop interviewing and skip to the next house.

Name of the interviewer ____________ Signature__________ Date of interview____

Name of the supervisor ______________Signature __________Date_____________
